# Limb-Clasping Response in NMDA Receptor Palmitoylation-Deficient Mice

**DOI:** 10.1007/s12035-024-04166-9

**Published:** 2024-04-09

**Authors:** Nami Suzuki, Akiko Oota-Ishigaki, Toshie Kaizuka, Masayuki Itoh, Maya Yamazaki, Rie Natsume, Manabu Abe, Kenji Sakimura, Masayoshi Mishina, Takashi Hayashi

**Affiliations:** 1 Biomedical Research Institute, National Institute of Advanced Industrial Science and Technology (AIST), Central 6 (6-10), 1-1-1 Higashi, Tsukuba, Ibaraki 305-8566 Japan; 2 National Center of Neurology and Psychiatry (NCNP), National Institute of Neuroscience, Kodaira, Tokyo 187-8502 Japan; 3 Department of Cellular Neurobiology, Brain Research Institute, Niigata University, Niigata, 951-8585 Japan; 4 Department of Animal Model Development, Brain Research Institute, Niigata University, Niigata, 951-8585 Japan; 5 Department of Molecular Neurobiology and Pharmacology, Graduate School of Medicine, University of Tokyo, Tokyo, 113-0033 Japan; 6 Brain Science Laboratory, The Research Organization of Science and Technology, Ritsumeikan University, Kusatsu, Shiga 525-8577 Japan

**Keywords:** NMDA receptor, GluN2B, Palmitoylation, Tyrosine phosphorylation, Excitatory synapses, Clasping response

## Abstract

Proper regulation of *N*-methyl-d-aspartate-type glutamate receptor (NMDA receptor) expression is responsible for excitatory synaptic functions in the mammalian brain. NMDA receptor dysfunction can cause various neuropsychiatric disorders and neurodegenerative diseases. Posttranslational protein *S*-palmitoylation, the covalent attachment of palmitic acid to intracellular cysteine residues via thioester bonds, occurs in the carboxyl terminus of GluN2B, which is the major regulatory NMDA receptor subunit. Mutations of three palmitoylatable cysteine residues in the membrane-proximal cluster of GluN2B to non-palmitoylatable serine (3CS) lead to the dephosphorylation of GluN2B Tyr1472 in the hippocampus and cerebral cortex, inducing a reduction in the surface expression of GluN2B-containig NMDA receptors. Furthermore, adult GluN2B 3CS homozygous mice demonstrated a definite clasping response without abnormalities in the gross brain structure, other neurological reflexes, or expression levels of synaptic proteins in the cerebrum. This behavioral disorder, observed in the GluN2B 3CS knock-in mice, indicated that complex higher brain functions are coordinated through the palmitoylation-dependent regulation of NMDA receptors in excitatory synapses.

**Supplementary Information:**

The online version contains supplementary material available at 10.1007/s12035-024-04166-9.

## Introduction

Glutamate is the predominant excitatory neurotransmitter in the mammalian brain. The *N*-methyl-d-aspartate (NMDA)-type glutamate receptor (NMDA receptor) plays a crucial role in synaptic plasticity and synaptogenesis, serving as a basis of higher brain functions [1–4]. NMDA receptors consist of three families of subunits: GluN1, GluN2, and GluN3 (also called NR1/GluRζ1, NR2/GluRε, and NR3) [5–8]. Among the multiple subtypes, the major functional NMDA receptors are heteromeric GluN1/GluN2 complexes with primary physiological relevance [9–12]. The glutamate-binding GluN2 subunits are composed of four gene members, GluN2A, GluN2B, GluN2C, and GluN2D (also known as NR2A–D or GluRε1–4). GluN2 subunits determine the functional and spatiotemporal diversity of GluN1/GluN2-containing NMDA receptors. The glycine-binding GluN1 subunit is ubiquitously expressed in neurons throughout the brain, whereas the different GluN2 subunits have more restricted patterns of distribution [5, 13]. In particular, GluN2B-containing NMDA receptors are expressed in murine brains from the embryonic period to adulthood and their expression fluctuates [14]. Moreover, GluN2B is crucial for the ion channel properties of functional NMDA receptors, postsynaptic macromolecular organization [15], and ethanol sensitivity [16].

Bidirectional changes in synaptic excitability are regulated by reversible posttranslational protein modifications of NMDA receptors, such as palmitoylation and depalmitoylation in glutamatergic synapses [17, 18]. GluN2A and GluN2B are dynamically palmitoylated on two distinct cysteine clusters in their comparably long C-terminal region, whereas the essential subunit, GluN1, has no palmitoylation sites in the intracellular region [19–22]. Palmitoylation and depalmitoylation of the cysteine cluster in the juxta-membrane region of GluN2B (Cys cluster I: Cys849, Cys854, and Cys871) control the stable synaptic expression and internalization of surface NMDA receptors, respectively. GluN2 depalmitoylation of the Cys cluster I regulates the constitutive endocytosis of NMDA receptors during synaptic development, most likely by preventing the Src family protein tyrosine kinase (PTK) Fyn-dependent phosphorylation of tyrosine-based internalization motifs in the GluN2 C-terminal region. GluN2B contains a consensus binding motif (YXXΦ, Φ represents a large hydrophobic residue) for the μ2 subunit of the clathrin adaptor protein-2 complex in its C-terminus (Y1472EKL). The activity-dependent phosphorylation of GluN2B at Tyr1472 by Fyn PTK suppresses clathrin-mediated endocytosis of surface NMDA receptors [23, 24]. Fyn-dependent phosphorylation of Tyr1472 is also required for the proper membrane localization of GluN2B-containing NMDA receptors at synapses in the hippocampus [25] and amygdala [26].

The dysfunction of NMDA receptors is associated with several neuropsychiatric disorders and neurodegenerative diseases [27–33]. Especially, GluN2B palmitoylation is involved in enhanced extrasynaptic surface expression of GluN2B-containing functional NMDA receptors, susceptibility to NMDA-induced toxicity in GABAergic striatal neurons [34], and steroid sensitivity [35]. Here, we report a limb-clasping response caused by impaired palmitoylation of the NMDA receptor. GluN2B juxta-membrane palmitoylation-deficient mice exhibited reduced GluN2B phosphorylation at Tyr1472 in the cerebrum and thus abnormal behavior.

## Materials and Methods

### Generation of Palmitoylation-Deficient GluN2B 3CS Mice and Experimental Animals

To construct the targeting vector, we identified a bacterial artificial chromosome (BAC) clone RP23-133P12 (BACPAC Resources Center) prepared from the C57BL/6 strain containing exon 11 and 12 of GluN2B using basic local alignment search tool searches against the mouse genome sequence database. The targeting vector comprised 5’-side and 3’-side arms with PGK–neomycin-resistant (neo) flanked by two loxP sites and the diphtheria toxin gene (DT) in which intron 11 was deleted. The 3’-side arm contained exon 11 with two palmitoylation sites Cys849 and Cys854 mutated to serines and exon 12 with a palmitoylation site Cys871 mutated to serine (three cysteines to serines: 3CS). In addition, a silent NcoI site mutation was introduced just after the serine mutation site to distinguish the mutant and wild-type (wt) alleles. All mutation procedures were performed by QuikChange II site-directed mutagenesis kit (Stratagene). These fragments were introduced into the pDEST-DT by Red/ET recombination using a BAC subcloning kit (Gene Bridges). The targeting vector was linearized by NotI and electroporated into embryonic stem (ES) cell line RENKA derived from the C57BL/6N strain, as described previously [36]. Then, G-418-resistant clones were picked as positive clones and confirmed by Southern blot hybridization analysis using outer probes. PCR-amplified 308 and 350 bp fragments and 635 bp PstI fragments from pLFNeo were used as 5’, 3’, and neo probes, respectively. The 5’ and 3’ probes were amplified by PCR using 5’-AATACAATGAAACCTTGCAAG-3’ and 5’-GTCATCAAAATTCAGAGCTTC-3’, 5’-AGAGCATGAAGGATCAGAATG-3’ and 5’-CCATCTCTGAAACCTGTCTTC-3’ as primers, respectively. Recombinant ES cells were injected into eight-cell stage embryos of the CD-1 mouse strain. The embryos were cultured to blastocysts and transferred to a pseudopregnant CD-1 mouse uterus. The resulting chimera mice were mated to C57BL/6N mice to yield heterozygous [GluN2B wt/3CS–Neo( +)] mice. These mice were crossed to CAG-*Cre* (backcrossed six times to C57BL/6) to remove the *neo* cassette from the germline through *Cre*/loxP-mediated excision. After confirmation of *neo* excision by Southern blot analysis and PCR, homozygotes 3CS/3CS mice were obtained by crossing heterozygous GluN2B wt/3CS pairs. Mutants with no *Cre* gene were used for subsequent breeding. The non-palmitoylation mutant mice were backcrossed onto the C57BL/6N strain at least three times. The GluN2B 3CS allele was identified by PCR using the primers 5’-CTGGAAGCTCTCTGGCTCAC-3’ and 5’-CGGTGGTGATGGTGATAGTG-3’. C57BL/6N and BALB/c mice were obtained from Charles River Laboratories. CAG-*Cre* mice were a kind gift from Dr. J. Miyazaki. Mice were fed with standard laboratory chow and water in standard animal cages under a 12-h light/dark cycle.

### Antibodies

Anti-GluA1 (ab31232, Abcam), anti-GluA2/3 (07–598, Millipore), anti-GluN1 (ab17345, Abcam), anti-GluN2A (AB1555P, Millipore), anti-GluN2B (ab65783, Abcam), anti-GluN2BpY1472 (cst 4208, Cell Signaling Technology), anti-PSD-95 (MA1-045, Thermo Fisher Scientific), anti-SAP102 (A7R8L, cst47421, Cell Signaling Technology), and anti-GAPDH (D16H11, cst5174, Cell Signaling Technology) antibodies were used for the experiments.

### Biochemical Analysis

Hippocampi or cortices were directly lysed in the SDS sample buffer. Lysates were separated by SDS-PAGE followed by Western blotting with each antibody. Reactive bands were visualized with the ECL Prime Western Blotting Detection System and chemiluminescent images were acquired using the ImageQuant LAS 4000 mini imager (GE Healthcare). Palmitoylation of NMDA receptor subunits and scaffolding MAGUK proteins were assessed using the acyl-biotinyl exchange (ABE) method, as previously described [37, 38]. Briefly, brain samples were directly denatured in lysis buffer containing 25 mM HEPES, pH 7.4, 150 mM NaCl, 2% SDS, 5 mM EDTA, protease inhibitor mixture (Roche), and 20 mM methyl methanethiosulfonate (MMTS) to block free thiols and acetone precipitation was used to move between steps. Following lysis, excess MMTS was removed by acetone precipitation and pellets were resuspended in a buffer containing 4% (w/v) SDS buffer (4SB). Samples were diluted and incubated for 1 h in either 0.7 M hydroxylamine (NH_2_OH, pH 7.4: (+)HA) to cleave thioester bonds or 50 mM Tris, pH 7.4 ((-)HA). After acetone precipitation to remove hydroxylamine or Tris, pellets were resuspended in 4SB and incubated for 1 h in 50 mM Tris, pH 7.4, containing 0.2 mM sulfhydryl-reactive biotin-HPDP (Cayman Chemical) at room temperature. To remove unreacted HPDP–biotin, acetone precipitations were performed and pellets were resuspended in 4SB. SDS was then diluted to 0.1% (w/v) and biotinylated proteins in the samples were affinity purified using Streptavidin Mag Sepharose (GE Healthcare). SDS sample buffer was used to cleave HPDP–biotin and to release purified proteins from the beads. The released proteins in the supernatant were denatured in the SDS sample buffer and processed for Western blotting. Background signals measured in (-)HA were subtracted from (+)HA signal intensities to accurately assess the 3CS mutation-induced palmitoylation changes.

### Histology

Brains were perfused and fixed with 4% paraformaldehyde (PFA) in PBS and transferred to 10%, 20%, and 30% sucrose in PBS solution step by step every 24 h. After tissues were embedded in optimal cutting temperature (OCT) compound, cryosections were cut at 20 μm thickness unless otherwise noted. Coronal sections were stained with cresyl violet and observed under the light microscope for Nissl staining.

### Neurological Screen

Neurological screening tests were performed as previously described [39–41]. The righting reflex, whisker twitch reflex, and ear twitch reflex were assessed on the first day. Mice were then placed in the box and the reflex response to key jangling, the auditory stimulus, was observed on the second day. Each mouse was tested three times. A number of physical features, including the presence of whiskers or fur condition, were also recorded.

### Assessment of Hind Limb–Clasping Behavior

Hind limb*–*clasping mice were suspended by the tail for 20 s to elicit the clasping phenotype, which was scored on a scale from 0 to 3, where 0 represented no limb clasping and normal escape extension; 1, one hind limb exhibited incomplete splay and loss of mobility; 2, both hind limbs exhibited incomplete splay and loss of mobility; 3, both hind limbs exhibited clasping with immobility [42–44].

### Experimental Design and Statistical Analysis

The design of each experiment and the statistical analysis methods used were described in each of the experimental sections. Statistical analyses were performed using Prism 9 (GraphPad Software) and Excel (Microsoft). All data are expressed as mean otherwise.

## Results

### Generation of GluN2B C-Terminal Palmitoylation Mutant Mice

**Fig. 1:**
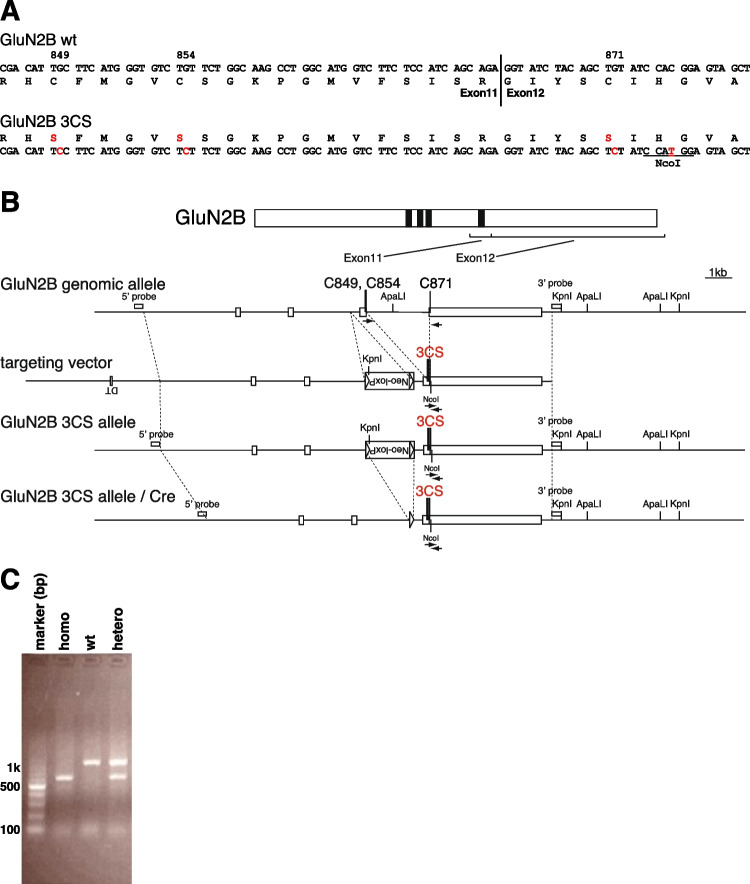
Generation of GluN2B C-terminal juxta-membrane palmitoylation-deficient mice. **A** GluN2B C-terminal juxta-membrane 3CS mutation and a silent NcoI site. Triple serine substitutions for Cys849, Cys854, and Cys871 and the silent NcoI site used to distinguish mutations are shown. **B** Top: Schematic representation of the GluN2B protein structure. Black bars indicate four transmembrane domains. Exons 11 and 12, encoding the C-terminal juxta-membrane palmitoylation sites (C849, C854, and C871), are shown. Middle: Genomic DNA structure with relevant restriction enzyme sites: ApaLI, KpnI. White boxes represent exons 9, 10, 11, and 12. Bottom: Targeting vector and targeted genes. The triple Ser substitutions at C849, C854, and C871 (3CS) are marked in red. Neo-loxP, phosphoglycerate kinase (PGK) promoter neomycin-resistance gene with the loxP sequence on both sides; arrows, PCR primers; silent NcoI site. The 5’ and 3’ probes for Southern blot analysis, which contain the genomic sequence outside of the targeting vector, are depicted as white bars. **C** PCR analysis of mouse tail samples from GluN2B 3CS homozygote (homo), wild-type (wt), and GluN2B 3CS heterozygote (hetero)

To elucidate the role of GluN2 palmitoylation in the regulation of synaptic function in vivo, we analyzed a line of GluN2B non-palmitoylation mutant mice lacking the C-terminal juxta-membrane palmitoylation site [20]. Three mutations at Cys849, Cys854, and Cys871 (three Cys to Ser: 3CS) in the Cys cluster I of GluN2B were introduced in the mice using homologous knock-in recombination techniques (Fig. 1A). A targeting vector encoding 3CS substitutions at Cys849 and Cys854 in exon 11 and Cys871 in exon 12 was constructed with a loxP-flanked *neo* marker in intron 10 (Fig. 1B). Correctly targeted embryonic stem (ES) cells containing the 3CS mutation and *neo* cassette were injected into blastocysts. Chimera mice carrying the mutant allele were bred with C57BL/6N mice to generate heterozygous mice [GluN2B wt/3CS-Neo( +)]. Heterozygous mice were then bred with CAG-*Cre* mice to delete the *neo* cassette from the germline via the *Cre*-loxP system (GluN2B wt/3CS) and intercrossed to produce homozygous mice (GluN2B 3CS/3CS, hereinafter called CS/CS). The success of the aforementioned procedures was confirmed by Southern blotting and polymerase chain reaction (PCR) analysis (Fig. 1C).

**Fig. 2:**
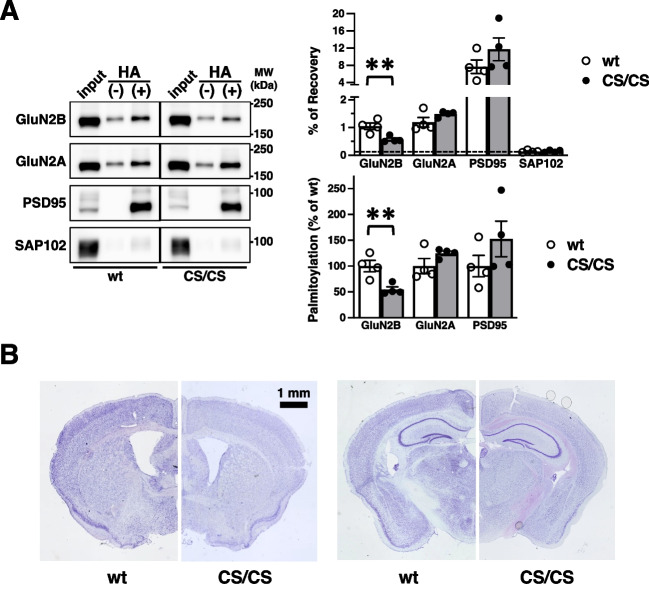
Reduced GluN2B palmitoylation and normal gross brain structure in GluN2B 3CS CS/CS mice. **A** Palmitoylation of synaptic proteins in GluN2B 3CS CS/CS mice. Representative blots of protein expression (input) and palmitoylation in whole-brain lysates prepared from wt and homologous GluN2B 3CS (CS/CS) mice, treated with ( +) or without (-) hydroxylamine (HA), demonstrate that the palmitoylation of GluN2B, but not of other proteins, was specifically reduced in CS/CS mice (left, *n* = 4). Percentage of recovery (right, top) and normalized levels to wt mice (right, bottom). White bars represent wt mice and dark bars represent CS/CS mice. Error bars indicate SEM. **p < 0.01, *t* test. **B** Macroscopic brain structure. Nissl-stained typical coronal sections across the caudate putamen (left) and hippocampus (right) from wt (comparable left half) or CS/CS (comparable right half) mice displayed no gross abnormalities in the cytoarchitecture. Scale bar, 1 mm

We initially confirmed the palmitoylation site mutation using ABE assay with specific antibodies (Fig. 2A). Decreased levels of GluN2B palmitoylation were detected in the whole brain of CS/CS mice (54.5 ± 5.1% compared with wt control, *n* = 4 mice, respectively; *p* = 0.0094; *t* test; Fig. 2A). The residual signals likely represented palmitoylation at Cys1215, Cys1218, Cys1239, Cys1242, and Cys1245 on the Cys cluster II in the middle of C-terminus, which was intact in CS/CS mice. The palmitoylation of other NMDA receptor GluN2A subunit (124.8 ± 4.2% compared with wt control, *n* = 4 mice, respectively; *p* = 0.1547; *t* test) and the major synaptic scaffolding protein, PSD-95 (152.6 ± 34.5% compared with wt control, *n* = 4 mice, respectively; *p* = 0.2382; *t* test), were unaffected in CS/CS mice, which ensured that GluN2B palmitoylation at juxta-membrane site was specifically absent without affecting other palmitoylated synaptic proteins. A non-palmitoylated synaptic scaffolding protein SAP102 was used to determine the background level of palmitoylation. Nissl-stained brain sections from adult CS/CS mice did not display any gross abnormalities in cytoarchitecture compared to those from wt mice (Fig. 2B).

### Mortality in the Juvenile Stage

**Fig. 3:**
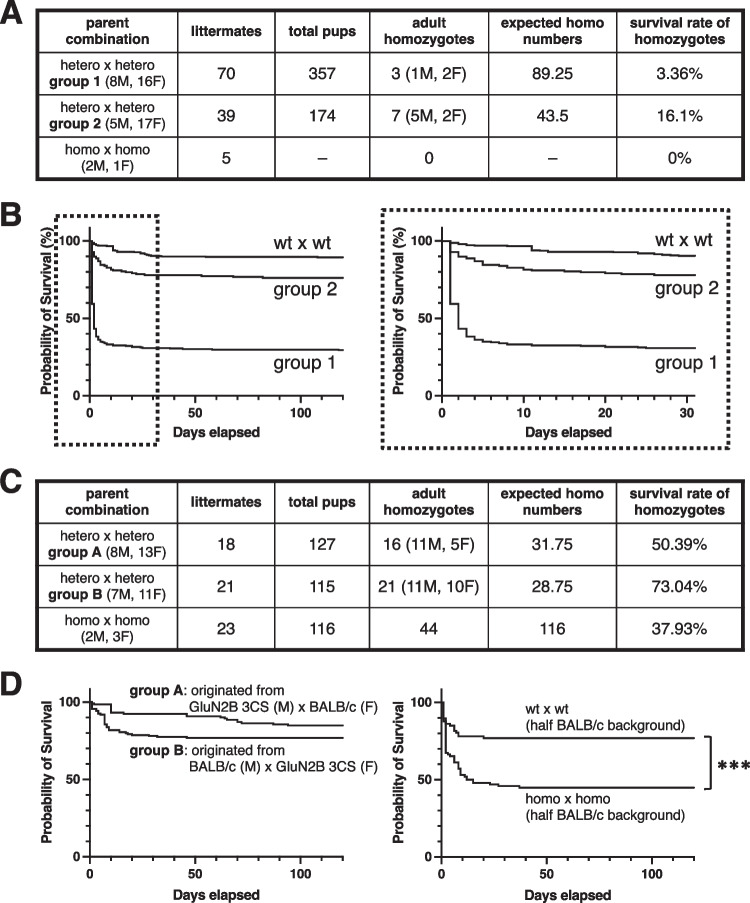
Low survival rates of GluN2B 3CS mice. **A** Intercrossing between GluN2B 3CS heterozygotes and homozygotes in a C57BL/6N-genetic background. Littermates, number of birth groups; total pups, number of total born pups appearing in cages; adult homozygotes, number of homozygotes grown to adulthood; expected homo numbers, quarter of total pups; survival rate of homozygotes, adult homozygotes/expected homo numbers. **B** Survival rate of GluN2B 3CS mutant mice with the C57BL/6N-genetic background (left, 4 months; right, 30 days). **C** Intercross of GluN2B 3CS heterozygotes and homozygotes in half C57BL/6N and half BALB/c genetic backgrounds. Descendants from initial founders between C57BL/6N-genetic background GluN2B 3CS males and BALB/c females (group A), and those from initial founders between BALB/c males and C57BL/6N-genetic background GluN2B 3CS females (group B). Littermates, number of births; total pups, number of total born pups appearing in cages; adult homozygotes, number of homozygotes grown to adulthood; expected homo numbers, quarter of total pups; survival rate of homozygotes, adult homozygotes/expected homo numbers. **D** Survival rate of GluN2B 3CS mutant mice in half C57BL/6N and half BALB/c genetic backgrounds (left, intercross of heterozygotes in groups A and B; right, inbred of wt or GluN2B 3CS homozygotes). ***p < 0.001, log-rank test

Theoretically, the intercross of heterozygotes without any pathological factors resulted in the production of wt, heterozygous, and homozygous offspring at the expected 1:2:1 Mendelian ratio. Most weak pups from the intercross of GluN2B 3CS heterozygotes with a pure C57BL/6N-genetic background died within 10 days. Only three homozygotes (one male, two female) in the first group of 357 pups, including 70 littermates born from 16 female heterozygotes, and seven homozygotes (five male, two female) in the second group of 174 pups, including 39 littermates born from 17 female heterozygotes, were confirmed by genotyping after weaning (Fig. 3A). No pups lived for more than 2 days among the five littermates born from the intercross of homozygotes. The survival rate of GluN2B 3CS mutant mice before weaning depended on the breeding environment (Fig. 3B: group 1 in an air-conditioned breeding rack, group 2 and intercross of C57BL/6N wt on breeding shelves in a biobubble).

Descendants from the initial founders of GluN2B 3CS males and BALB/c females (Fig. 3C, group A), or vice versa (Fig. 3C, group B), displaying better survival rates than GluN2B 3CS with a pure C57BL/6N-genetic background, were used for further evaluation. When the offspring of heterozygous GluN2B 3CS mutant mice, which managed to survive beyond the juvenile mortality stage, were interbred with mice having half C57BL/6N and half BALB/c genetic backgrounds, they showed normal growth into adulthood, exhibited typical breeding behavior, and demonstrated similar fertility levels to wt mice of the same genetic background (Fig. 3D). The intercross of homozygous GluN2B 3CS mutant mice showed a lower survival rate than that of wt or heterozygotes but better than that of homozygotes with a pure C57BL/6N-genetic background.

### Augmented GluN2B Phosphorylation at Tyr1472 and Normal Synaptic Protein Expression in GluN2B 3CS Mutant Mice

**Fig. 4:**
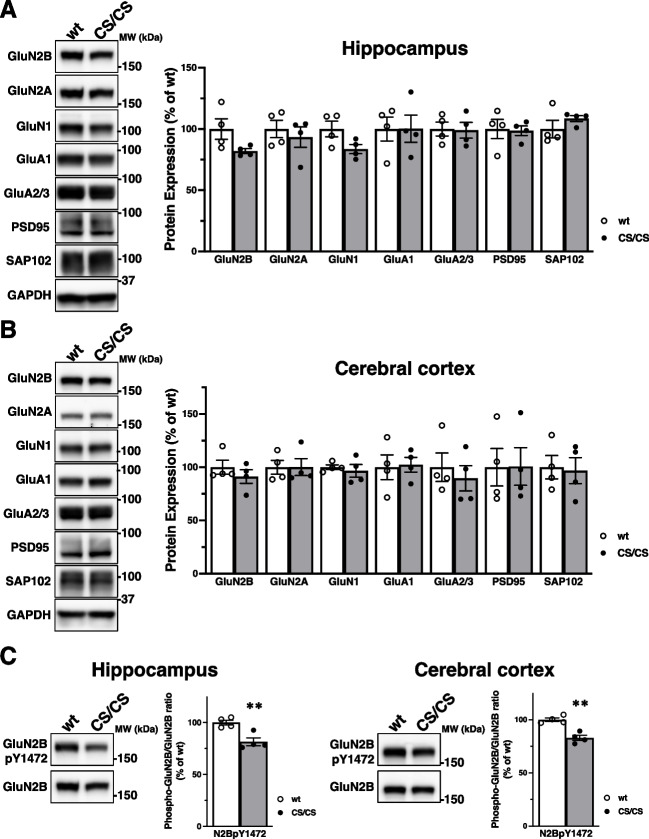
Protein expression and phosphorylation of synaptic proteins in the hippocampus and cortex of GluN2B 3CS mutant mice. **A** Protein expression levels of NMDA receptor subunits, AMPA receptor subunits, PSD-95, and SAP102 in hippocampal lysates from wt (white bars) and CS/CS (dark bars) mice. Typical representative blots (left) and protein expression levels are shown (right, wt, *n* = 4; CS/CS, *n* = 4; GluN2B, *p* = 0.082; GluN2A, *p* = 0.564; GluN1, *p* = 0.068; GluA1, *p* = 0.989; GluA2/3, *p* = 0.906; PSD-95, *p* = 0.875; SAP102, *p* = 0.301). **B** Protein expression levels of NMDA receptor subunits, AMPA receptor subunits, PSD-95, and SAP102 in cortical lysates from wt (white bars) and CS/CS (dark bars) mice. Representative blots (left) and protein expression levels are illustrated (right, wt: *n* = 4; CS/CS: *n* = 4; GluN2B, *p* = 0.377; GluN2A, *p* = 0.564; GluN1, *p* = 0.615; GluA1, *p* = 0.867; GluA2/3, *p* = 0.582; PSD-95, *p* = 0.978; SAP102, *p* = 0.853). **C** GluN2B phosphorylation at Y1472 in the hippocampus and cerebral cortex of wt (white bars) and CS/CS (dark bars) mice. Typical blots are displayed as representative samples (left). Phosphorylation levels normalized to GluN2B protein expression are shown (right) (wt: *n* = 4; CS/CS: *n* = 4). Error bars indicate SEM. **p < 0.01, *t* test

Next, we examined the protein expression and phosphorylation of the NMDA receptor subunits GluN1, GluN2A, and GluN2B; the major α-amino-3-hydroxy-5-methyl-4-isoxazole propionate (AMPA) receptor subunits, GluA1, GluA2, and GluA3; PSD-95 and SAP102 in the adult hippocampus (Fig. 4A) and cerebral cortex (Fig. 4B). Western blot analysis showed no significant difference in the expression of these synaptic proteins between wt and CS/CS mice. The expression level of GluN2B and GluN1 trended towards a decrease in the hippocampus of CS/CS mice, but this was not statistically different (Fig. 4A; GluN2B: 82.0 ± 2.0% compared with wt, *n* = 4 mice, respectively, *p* = 0.082, *t* test; GluN1: 83.6 ± 3.7% compared with wt, *n* = 4 mice, respectively, *p* = 0.068, *t* test). Furthermore, GluN2B phosphorylation was decreased at Tyr1472 (Y1472) in the adult hippocampus (Fig. 4C, left: 81.5 ± 3.8% compared with wt, *n* = 4 mice, respectively; *p* = 0.0062; *t* test) and cerebral cortex (Fig. 4C, right: 83.0 ± 2.5% compared with wt, *n* = 4 mice, respectively; *p* = 0.0014; *t* test) of CS/CS mice. GluN2B phosphorylation at Y1472 increases in a synaptic activity-dependent manner [45].

### Hind Limb–Clasping Response in GluN2B 3CS Mutant Mice

**Fig. 5:**
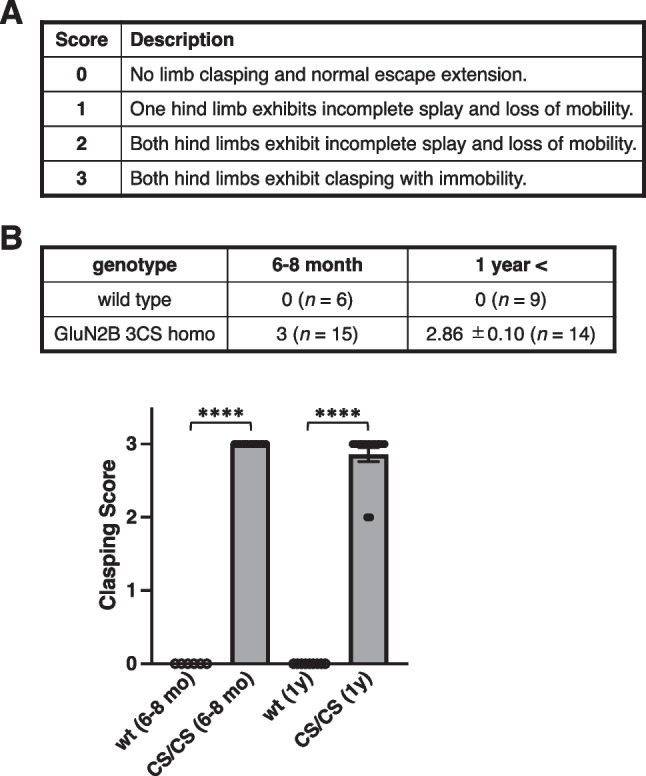
Hind limb–clasping response. **A** Description of scoring of hind limb clasping. **B** Clasping scores of 6–8 month or 1-year-old wild-type (wt) and GluN2B 3CS homo (CS/CS, dark bars) mutant mice. The average score and number of mice used in this experiment are shown. Error bars indicate SEM. ****p < 0.0001, two-way analysis of variance (ANOVA) followed by Tukey–Kramer multiple comparisons test

Rodents usually extend their limbs in anticipation of contact with stable ground or fixed scaffolding when they hang upside down by their tail, possibly triggered by visual or gravitational stimuli [46]. The commonly used C57BL/6 mice display better visual placement responses than other strains [42]. Abnormal responses such as limb clasping are induced by genetic fragility related to the neural pathways responsible for limb extension. To assess the signs of neurological deficits and behavioral impairments in GluN2B 3CS mutant mice, we tested the hind limb–clasping response (Fig. 5A). GluN2B 3CS mutant mice exhibited a loss of the normal hind limb extension reflex; meanwhile, 27 CS/CS mice exhibited severe clasping and two CS/CS mice demonstrated weak clasping responses (Fig. 5B). All wt mice with half C57BL/6N and half BALB/c genetic backgrounds produced a normal response. Concerning other sensorimotor responses, both wt and CS/CS mice displayed normal neurological reflexes (Supplementary Table).

## Discussion

We previously revealed that the reduction of GluN2B palmitoylation decreases phosphorylation of GluN2B Tyr1472 in cultured cortical neurons and thereby downregulates synaptic expression of GluN2B-containing NMDA receptors in organotypic hippocampal slice cultures [20, 22]. The present study provides further evidence supporting that GluN2B depalmitoylation is involved in behavioral changes. Neonatal GluN2B 3CS mutant mice displayed a lower survival rate than expected in healthy mice (Fig. 3). Juvenile death of feeble GluN2B 3CS pups is influenced by both feeding environment and genetic background. GluN2B protein expression peaks at around the first postnatal week [5, 13]. The underlying mechanism appears to be deficient sucking ability in these non-palmitoylated newborns, as observed in homozygous GluN2B-knockout mice [47]. The GluN2B-null mutant pups show severe defects in the sucking response and die shortly after birth, even if their mother nurse normally. The sucking response requires rhythmic coordination of the lips and oral movements, including nipple attachment, sucking with rhythmic movements of the jaw and tongue, and the stretch response [48]. These processes are regulated by complex interactions between the sensory and motor neuronal pathways. An abnormal reduction in synaptic GluN2B-containing NMDA receptors in sensory- and/or motor-related circuits causes this defect in GluN2B 3CS non-palmitoylation mutant pups. Another possibility is the low maternal breastfeeding ability of GluN2B 3CS homozygotes. Further studies are needed to address more detailed neural circuit responsible for this phenomenon.

Adult GluN2B 3CS homozygous mice exhibited a definite hind limb–clasping response during tail suspension (Fig. 5). Generally, this morbid phenotype is associated with abnormal motor responses related to various factors in the cerebellum, basal ganglia, and neocortex, including the cerebello-cortico-reticular and cortico-striato-pallido-reticular pathways [42]. After becoming an adult, GluN2B gene expression is eliminated from the cerebellum, suggesting that palmitoylatable GluN2B-expressing neurons in cortico-striato-pallido-reticular circuits are related to normal response [5, 13, 49]. Furthermore, our results demonstrated that the lack of GluN2B juxta-membrane palmitoylation sites did not affect the gross brain anatomy (Fig. 2B) or the general palmitoylation of other synaptic proteins (Fig. 2A). Normal expression of a series of synaptic proteins, NMDA receptors, AMPA receptors, and scaffolding proteins and significantly decreased phosphorylation of GluN2B Tyr1472 were detected in the hippocampus and cerebral cortex of GluN2B 3CS homozygotes (Fig. 4), suggesting that GluN2B expression on the synaptic surface was consistently downregulated in the cerebrum of GluN2B 3CS homozygotes. These data indicate that 3CS non-palmitoylation mutations reduce the surface expression of functional NMDA receptors [20, 22] and alter the localization of GluN2B-containing NMDA receptors on excitatory glutamatergic synapses [50] in the adult cerebrum. GluN2B 3CS homozygotes did not show any changes in other neurological reflexes (Supplementary Table). In addition, the mutant mice appeared to see around accurately because they normally reacted to the experimenter’s movements. These observations indicate that there is no 3CS mutation-induced widespread abnormality in their sensorimotor system and therefore GluN2B 3CS homozygotes can accept external stimuli. Together, the impaired motor response is possibly caused by deficits in palmitoylation-dependent regulation of GluN2B-containing NMDA receptors in specific cerebral synaptic functions.

Proper synaptic palmitoylation plays a crucial role in glutamatergic excitability, resulting in the maintenance of brain functions [17, 38, 41, 51, 52]. Palmitoylation sites in the Cys cluster I of evolutionally diverged GluN2B orthologs are almost completely conserved only in the vertebrate lineage [53, 54]. The abnormal behavior in palmitoylation-deficient GluN2B mice, as shown here, may be related to various neurological diseases in humans.

## Supplementary Information

Below is the link to the electronic supplementary material.

Supplementary file1 (PDF 31 KB)

## Data Availability

The data that support the findings of this study are available from the corresponding author upon reasonable request.
