# Supplementary material for: Limb-Clasping Response in NMDA Receptor Palmitoylation-Deficient Mice
Source: Mol Neurobiol. 2024 Apr 9;61(11):9125–35. doi: 10.1007/s12035-024-04166-9 (PMC11496324; doi:10.1007/s12035-024-04166-9)
Supplement: Supplementary file 1 — Supplementary file1 (PDF 31 KB) [file 12035_2024_4166_MOESM1_ESM.pdf]

Supplementary Table. General physical characteristics and sensory functions

	wild type (n = 6)	GluN2B 3CS homo (n = 15)
Physical characteristics	100	100
whiskers (% with)	100	100
fur (% with normal fur)	100	100
Sensory motor reflex	100	100
righting reflex (% with normal response)	100	100
whisker twitch reflex (% with normal response)	100	100
ear twitch reflex (% with quick response)	100	100
reflex response to key jangling (% with normal response)	100	100
